# In the Eye of the Storm: Bi-Directional Electrophysiological Investigation of the Intact Retina

**DOI:** 10.3389/fnins.2022.829323

**Published:** 2022-02-25

**Authors:** Ieva Vėbraitė, Yael Hanein

**Affiliations:** ^1^School of Electrical Engineering, Tel Aviv University, Tel Aviv, Israel; ^2^Tel Aviv University Center for Nanoscience and Nanotechnology, Tel Aviv University, Tel Aviv, Israel

**Keywords:** retina, multi electrode arrays, neurostimulation, micro-electrodes, soft neural interfaces, bi-directional electrophysiology

## Abstract

Electrophysiological investigations reveal a great deal about the organization and function of the retina. In particular, investigations of explanted retinas with multi electrode arrays are widely used for basic and applied research purposes, offering high-resolution and detailed information about connectivity and structure. Low-resolution, non-invasive approaches are also widely used. Owing to its delicate nature, high-resolution electrophysiological investigations of the intact retina until now are sparse. In this Mini Review, we discuss progress, challenges and opportunities for electrode arrays suitable for high-resolution, multisite electrophysiological interfacing with the intact retina. In particular, existing gaps in achieving bi-directional electrophysiological investigation of the intact retina are discussed.

## Introduction

Non-invasive electrophysiological interfaces for the retina are important in the study of the retina and the visual system. Non-invasive electrophysiological investigations are widely used both for medical and research purposes (Kremers et al., 2020). Primarily, electroretinogram (ERG) is used for low-resolution clinical diagnostics of the retina, but may also be applied to explore brain disorders, as few recent studies have suggested. Non-invasive ERG employs electrodes placed in contact with the cornea to record retinal potentials in response to visual stimuli (e.g., flash, pattern, or multifocal) (Frishman, 2009). As the retina is an anatomical extension of the central nervous system (CNS), it may serve as a window to the brain (Meister and Berry, 1999), and retinal function may reflect neurological dysfunctions in psychiatric disorders, such as schizophrenia, multiple sclerosis, and Parkinson’s disease, to name three examples (Schwitzer et al., 2017). Retinal ganglion cell dysfunction is also apparent in preclinical Alzheimer’s disease (Asanad et al., 2021). ERG could also help in differentiating multiple sclerosis from neuromyelitis optica spectrum disorder, and schizophrenia from bipolar disorder (Filgueiras et al., 2019; Hébert et al., 2020). In these examples, a non-invasive electrophysiological study using ERG provided telltale information about both retina and brain conditions.

High-resolution *ex vivo* investigation of the retina is another important example of the utility of electrophysiology in the study of the retina. It is widely applied to understand neural processes in retina development, function, and degeneration. Spontaneous retinal waves, as an example, is a electrophysiological phenomena of the developing retina (Wong, 1999). Waves are hypothesized to contribute to the formation of proper connectivity of the nervous system and can be readily observed and studied *ex vivo*. Spontaneous waves generally disappear after eye opening, but they become evident again during retinal degeneration. It was shown, for example, that explanted retinas of mice models of retinal degeneration (i.e., rd1 and rd10) have spontaneous bursts at 100–240 ms intervals, similar to that observed in the developing retina (Demas et al., 2003; Stafford et al., 2009; Goo et al., 2011). On the other hand, spiking frequency within bursts was observed to be higher in the developing retina (above 10 Hz) (Stafford et al., 2009). This spontaneous spiking activity in the form of bursts and waves may have important applied implications as they may interfere with vision restoration strategies, as we further discuss below (Goo et al., 2016; Haselier et al., 2017; Im and Kim, 2020). *Ex vivo* investigations are used to study many other important electrophysiological phenomena in the retina, such as intrinsically photosensitive ganglion cells (ipRGC) responses to light stimulation, and retina cell (ganglion or bipolar) responses to electrical stimulation, to name just two examples (Sekhar et al., 2017; Im et al., 2018; Mure et al., 2019).

**FIGURE 1:**
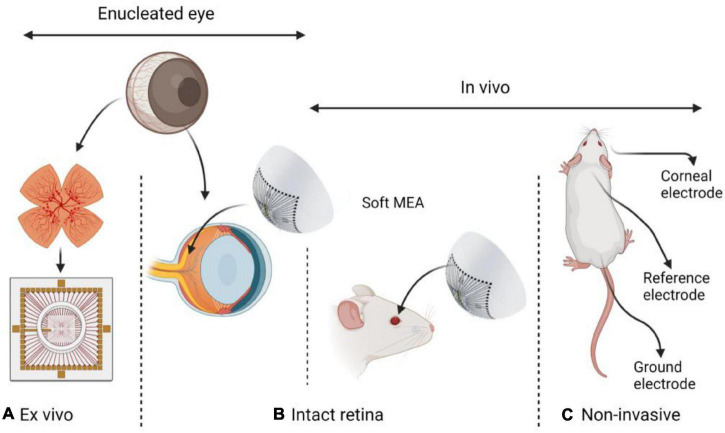
Different approaches in retinal electrophysiology research: **(A)** *Ex vivo* – explanted retina. **(B)** Intact retina either in an enucleated eye or *in vivo*. **(C)** Non-invasive ERG *in vivo*. Common animal models include rabbits, rats, mice, chick embryos, and pigs. While *ex vivo* investigations require eye enucleation and are performed either with intact or explanted retina, *in vivo* investigations are performed on an intact retina in a living animal.

In contrast to the abundance of high-resolution *ex vivo* and non-invasive low-resolution electrophysiological studies of the retina, few multi-electrode electrophysiological investigations of the intact retinas were reported. Nevertheless, recent advances in soft electronics for neural interfacing offer new opportunities toward long-term high-resolution recordings and bi-directional interfacing with the intact retina. In this mini-review, we present examples of neural interfaces that were applied for the study of the intact retina. We further explain the opportunities in studying the electrophysiology of the intact retina, and present recent technological progress toward such investigations. In Figure 1, we illustrate the different electrophysiological approaches discussed in this Mini-review: *Ex vivo*, invasive and non-invasive methods.

## Multi Electro Arrays for Electrophysiological Study of the Intact Retina

An electrophysiological study of the intact retina can be achieved with electrodes mounted on micromanipulators. The electrodes are introduced into the eye and electrical recordings can be performed (Maffei and Galli-Resta, 1990). Using such a method, spontaneous discharges of neighboring retinal ganglion cells were recorded simultaneously in anesthetized prenatal rats, using glass micropipettes filled NaCl, which were lowered through the lens to reach the retina. Clearly, such a straightforward approach is limited and does not allow high-resolution, multisite recordings, nor the study of the retina in freely behaving animals. Devices suitable for high-resolution, multisite electrophysiological study of the intact retina clearly impose some of the most restricting requirements for neural interfacing. Owing to the technical challenges associated with electrophysiological investigations of the intact retina, attention was directed toward optimized flexible and soft electronics.

In one early study, flexible polyimide thin-film microelectrode arrays for retinal stimulation and recording were tested (Mathieson et al., 2013). The electrode array consisted of Pt electrodes 5 μm in diameter, embedded in 15 μm polyimide film. Light and electrically evoked retinal responses were recorded from an intact frog retina.

**FIGURE 2:**
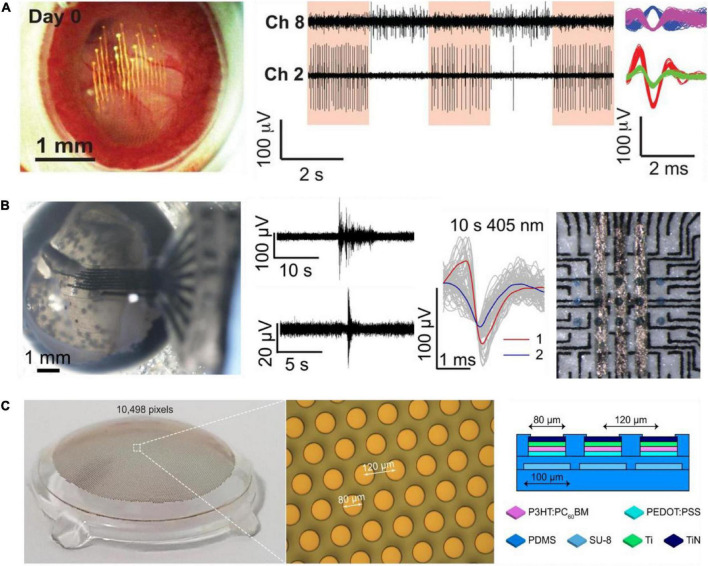
Soft electrode arrays for electrophysiological studies in the intact retina. **(A)** Image of mesh electronics inside a mouse eye (left). RGC responses to light modulation recordings on day 14 after injection (middle). Sorted spikes assigned to different neurons (right). Adapted with permission from Hong et al. (2018). **(B)** Image of soft carbon probe placed inside an enucleated chick eye (left). Recorded spontaneous activity and responses to light stimuli (middle). Soft carbon MEA with organic photo-capacitors (230 mm) for bi-directional electrophysiology. Adapted from Vėbraitė et al. (2021). **(C)** Wide field photovoltaic retinal prosthesis. Adapted from Chenais et al. (2021b). Copyright© 2021, The Author(s).

More recently, *in vivo* recordings from RGCs in awake mice, using epi-retinal-implanted mesh electronics, was reported (Figure 2A; Hong et al., 2018). The study presented recordings of circadian rhythms in RGC and RGC responses to visual stimuli. The recording device consisted of Cr/Au metal interconnect lines and 16 Pt recording electrodes, 20 μm in diameter on SU-8 polymer. In a follow-up study, the mesh electrodes were adapted to accommodate 32 recording electrodes and were used to study mouse RGC activity and responsiveness changes after an optic nerve crush (ONC; Tran et al., 2019). The use of the SU-8 based mesh is an important enabling technology, which allows the injection of the device to the eye, along with high conformity with the retina curvature. High-quality recordings of retinal activity, in particular individual RGCs recording for a period of 2 weeks, were demonstrated *in vivo*.

Soft devices suitable for retinal stimulation are also emerging. For example, a stimulating photo-capacitor on soft and thin (20 mm) polyurethane (PU) film was recently demonstrated in Vėbraitė et al. (2021; Figure 2B). Such photo-capacitors comprised of p-n semiconducting organic pigments charge up under 660 nm illumination and generate displacement currents that elicit retinal responses (Rand et al., 2018). Screen printed carbon electrodes (60 μm in diameter, spaced 520 μm apart) enabled simultaneous recording by the same device, as well as allowed identification of neuronal units. Plasma polymerized 3,4-Ethylenedioxythiophene (EDOT) was used to improve electrode specific capacitance, and the electrical recording of neurons in the retina were achieved both *ex vivo* and in the retina still in the enucleated eye of chick embryos. Probes consisting of eight electrodes were used for recording in the intact retina. Using these soft electrodes, it was possible, for the first time, to observe spontaneous multi-site waves in the intact developing chick retina (embryonic day 13), with properties similar to those observed in the explanted retina.

Recently, Chenais et al. (2021b) presented a soft photovoltaic retinal stimulation device suitable for wide-field retina stimulation. The stimulating device includes 10,498 pixels (80 μm in diameter) on a PDMS substrate covering a 43° visual angle (Figure 2C). The study demonstrated high spatial resolution stimulation of retinal ganglion cells. These soft devices for retina stimulation were not yet integrated with recording electrodes and tested in the intact retina.

## What Can Be Further Gained From a Bidirectional Communication With the Intact Retina?

The study of the retina in its explanted form is associated with several limitations which may be overcome by recording from the intact retina. Primarily, explanted retinas lack the metabolic environment of the eye, and some of its electrophysiological properties under *ex vivo* conditions may be distorted. For example, identifying stimulation parameters for retinal implant performance optimization may depend on retina metabolic conditions (Ingensiep et al., 2021) and retina explant viability (Balasubramanian and Shabanian, 2020). Therefore, *ex vivo* stimulation studies, for example, may provide only a partial picture of retinal response to electrical stimulation.

In the realm of artificial vision, bi-directional recording and stimulation may contribute to enhance the design of new devices and improved stimulation strategies (Shah and Chichilnisky, 2020). One major limitation of contemporary devices is the inability to record and stimulate with the same device. Retina responses to stimulation are often studied *ex vivo* in MEA configuration or with an external sharp metal electrode placed at the vicinity of individual RGCs (Chenais et al., 2021b). Montes et al. (2020) recently presented a new approach based on a penetrating device that can reach and stimulate at different depths inside the explanted retina and simultaneously record RGC spiking activity. In animal models and humans, stimulation efficacy can be estimated by recording visually evoked potentials (VEPs) in the visual cortex (Huang et al., 2021). In animal models, optogenetics can provide a non-invasive imaging of retinal responses at high resolution (Cheong et al., 2018). This novel technology may be used for long-term retinal function tracking.

Despite many years of investigations and technological development, retinal implants have still limited performances. Beyond many technical challenges, a remaining open question is how visual perception can be achieved beyond the perception of isolated phosphenes. The vast range of parameters (such as pulse duration frequency and amplitude) limits the ability to carry out such investigations on patients. For example, desensitization, the process in which visual perception is fading has been debated (Avraham et al., 2021; Chenais et al., 2021a). *Ex vivo* experiments provide limited insight regarding visual perception, as stimulation parameters may depend on physiological conditions. Also, due to unstable and weaker interface between the implant and the retina, frequent stimulation recalibration may be necessary (Shah and Chichilnisky, 2020). Recording from the retina, in conjunction with computational vision modeling and electrical stimulation, may help reveal proper stimulation parameters (Yu et al., 2020; Ghaffari et al., 2021), in particular for epi-retina approaches. Soft multi-electrode arrays, capable of bidirectional electrophysiological investigations in the intact retina may play an important role in resolving much of the ambiguity associated to stimulation efficacy and parameter optimization at the site of stimulation.

It is important to note that epi-retinal configurations have so far showed limited performances, and recent vision restoration strategies focus on sub-retinal or cortical stimulation. A prosthesis showing great results include subretinal implant PRIMA (Pixium Vision) and cortical prosthesis ORION (Second Sight Medical Products) (Palanker et al., 2020; Caspi et al., 2021). Nevertheless, epiretinal implants based on soft materials may still play an important role, in particular for peripheral wide field vision restoration, where a soft probe can conform to the curvature of the retina, and in improving the mechanical implant-retina interface.

The study of brain abnormalities through abnormal retina function is a relatively recent field which may also benefit from better interfacing with the intact retina. Non-invasive approaches have revealed several interesting insights. Multiple ERG studies showed retinal functionality anomalies (in a-wave and b-wave amplitudes and latencies, ON and OFF RGC responses) in schizophrenia, autism, bipolar disorder, multiple sclerosis and other psychiatric and neurodevelopmental disorders (Silverstein et al., 2020).

Another interesting domain which can gain from high-resolution electrophysiological interfacing with the retina is transorbital altering current stimulation (ACS). It was suggested that transcranial or transorbital ACS could be used to treat various psychiatric disorders or optic nerve conditions, respectively (Elyamany et al., 2021; Lee et al., 2021). High-resolution studies of the retina in animal models may help to understand these intriguing phenomena and to better map retinal function abnormality linked with psychiatric disorders by revealing the mechanism of action and optimal parameters for ACS.

The study of the circadian clock is yet another example for the possible benefits of bidirectional electrophysiology from the intact retina. There are indications that circadian clock disruption may be a factor in age-related macular degeneration incidence, behavioral health, and psychiatric problems (DeVera et al., 2019; Walker et al., 2020). Blue light sensitivity of the retina is readily apparent *ex vivo*. Ganglion cells with intrinsic light sensitivity can be optically stimulated and electrically measured. Studies investigating the effect of circadian disruption and ipRGC activity would provide more knowledge and suggest new treatment methods.

Finally, spontaneous waves in the retina have been studied extensively, however, questions regarding the validity of *ex vivo* model of the retina remain. For example, bursts occurred more frequently *in vivo* than in *ex vivo* retinal investigations (Hanganu et al., 2006). Is this difference due to the result of the artificial *ex vivo* conditions? The study of spontaneous waves in the intact retina will allow the investigation under more neutral conditions. Spontaneous waves or retina hyperactivity are also known to appear in retina degeneration. Whether and how such retinal activity affects retinal degeneration is interesting and can impact retina implant technology (Stasheff, 2018). Walter (2017) points out the need for recording and understanding spontaneous retinal activity close to the stimulating electrode and optimizing stimulation parameters accordingly. Investigation of waves in retina degeneration in the intact retina could provide not only insights, but could also confirm the low-frequency spontaneous waves with bursting spikes described by Haselier et al. (2017) in explanted retinas, as well as to verify electrical stimulation strategies to suppress such waves and successfully evoke phosphenes.

## If You Can Make It There You Can Make It Everywhere

Reports on flexible and soft neural electrodes for deep brain stimulation, sciatic, tibial, vagus nerve stimulation are abundant (Kleber et al., 2019; Obidin et al., 2019; Liu et al., 2020; Cho et al., 2021; Zhou et al., 2021). Under the definition of flexible and soft, we refer to substrate materials, such as polyimide, parylene C, PDMS and PU with Young’s modulus in the range of 0.002–4 GPa (Vėbraitė and Hanein, 2021). Recent studies have addressed many challenges associated with the fabrication of these devices, including their stability and electrode performances (Boehler et al., 2017). Fabrication strategies vary and include photo-lithography, laser cutting, ink jet printing, and screen printing, to name just a few examples. Electrode materials and deposition strategies also vary significantly. Nevertheless, most existing flexible and soft neural electrodes for electrical stimulation and recordings do not cover investigations from the intact retina. Most contemporary probes suffer from inferior electro-chemical properties (Vėbraitė and Hanein, 2021), resulting in lower resolution and poor probe stability, owing to adverse reactions between flexible materials and the physiological environment. Specifically, flexible polymers are inherently prone to cracks and water absorption. As a result, the quality of the electrodes on a flexible substrate is generally inferior to the state of the art in neural electrodes, hindering high resolution and high-quality long-term recording with these devices, as well as bi-directional interfacing. New strategies to fabricate soft neural probes continue to emerge (Wang et al., 2019; Dong et al., 2021) and some may prove useful for long-term bi-directional retinal interfacing.

## Discussion

Table 1 summarizes main electrophysiological approaches discussed in this mini-review. Owing to the rarity of *in vivo* high-resolution electrophysiological studies of the retina, much of what we know about the retina electrical activity is based on the explanted retina model. Explanted retinas of different animal models can be readily investigated *ex vivo* using high-resolution electrophysiology. In particular, multi electrode arrays are commonly used to study retina electrical activity during the developmental stages, degenerative diseases, or to investigate retina responses to electrical or optical stimulation, to name just a few examples. Recent progress toward more stealthy neural probes establishes important first steps toward the wider adoption of soft retinal interfaces. Finally, better devices that are suitable for bi-directional retinal interfacing will contribute to improved technology that can be used far and beyond retinal interfacing.

**TABLE 1:** Summary of retinal prosthesis discussed.

Device	Functionality	Device placement	References
Thin-film flexible microelectrode array	Recording and stimulation	*In vivo* Frog Epiretinal placement	Mathieson et al.,2013
Mesh electronics	Recording	*In vivo* Mice Epiretinal placement	Hong et al.,2018
SoftC probe	Recording With a possibility to stimulate with organic photo-capacitor pixels	*Ex vivo* – intact retina Chick embryo Epiretinal placement	Vėbraitė et al.,2021
POLYRETINA	Photovoltaic stimulation	*Ex vivo* – explanted retina Mice Epiretinal placement	Chenais et al.,2021b
Intraretinal probes	Recording and stimulation	*Ex vivo* – explanted retina Mice Different depths within the retina from RGC side	Montes et al.,2020
PRIMA (Pixium Vision)	Photovoltaic stimulation	*In vivo* Humans Subretinal placement	Palanker et al.,2020
ORION (Second Sight Medical Products)	Stimulation	*In vivo* Humans Cortical placement	Caspi et al.,2021

Owing to the challenging intra-ocular position and delicate nature of the retina, high-resolution devices for bi-directional recordings and stimulation from the intact retina are presently unavailable. As it was reviewed above, some soft electrodes with high resolution are beginning to meet the requirements needed for high-resolution electrophysiology in the intact retina. Moreover, soft neural interfaces that target the retina provide exciting opportunities in the investigation of retina structure and function. Such devices may play a role in a wide range of applications, ranging from basic investigations, to better understanding of artificial retina stimulation, to closed loop electrical stimulation of the retina.

## Author Contributions

All authors listed have made a substantial, direct, and intellectual contribution to the work, and approved it for publication.

## Conflict of Interest

The authors declare that the research was conducted in the absence of any commercial or financial relationships that could be construed as a potential conflict of interest.

## Publisher’s Note

All claims expressed in this article are solely those of the authors and do not necessarily represent those of their affiliated organizations, or those of the publisher, the editors and the reviewers. Any product that may be evaluated in this article, or claim that may be made by its manufacturer, is not guaranteed or endorsed by the publisher.
